# Methyl gallate and tylosin synergistically reduce the membrane integrity and intracellular survival of *Salmonella* Typhimurium

**DOI:** 10.1371/journal.pone.0221386

**Published:** 2019-09-06

**Authors:** Abraham Fikru Mechesso, Quah Yixian, Seung-Chun Park

**Affiliations:** Laboratory of Veterinary Pharmacokinetics and Pharmacodynamics, College of Veterinary Medicine, Kyungpook National University, Bukgu, Daegu, South Korea; INRA, FRANCE

## Abstract

*Nymphaea tetragona* Georgi (Nymphaceae) is traditionally used in Asia for the treatment of diarrhea, dysentery and fever. The plant contains various active compounds, including methyl gallate (MG) which are reported to inhibit bacterial virulence mechanisms. This study aimed to evaluate the alterations on viability, membrane potential and integrity of *Salmonella enterica* Serovar Typhimurium exposed to MG in combination with Tylosin (Ty), which is relatively inactive against Gram-negative bacteria, but it is commonly used as a feed additive in livestock. Besides, the effects of sub-inhibitory concentrations of the combination (MT) on the interaction between *S*. Typhimurium and the host cell, as well as on the indirect host responses, were characterized. Flow cytometry, confocal and electron microscopic examinations were undertaken to determine the effects of MT on *S*. Typhimurium. The impacts of sub-inhibitory concentrations of MT on biofilm formation, as well as on the adhesion, invasion and intracellular survival of *S*. Typhimurium were assessed. The result demonstrated significant damage to the bacterial membrane, leakage of cell contents and a reduction in the membrane potential when treated with MT. Sub-inhibitory concentrations of MT significantly reduced (*P* < 0.05) the biofilm-forming, adhesive and invasive abilities of *S*. Typhimurium. Exposure to MT drastically reduced the bacterial count in macrophages. Up-regulation of interleukin (IL)-6, IL-8 and IL-10 cytokine genes were detected in intestinal epithelial cells pre-treated with MT. This report is the first to describe the effects of MT against *S*. Typhimurium. The result indicates a synergistic interaction between MG and Ty against *S*. Typhimurium. Therefore, the combination may be a promising option to combat *S*. Typhimurium in swine and, indirectly, safeguard the health of the public.

## Introduction

*Salmonella enterica Serovar* Typhimurium is one of the causative agents of food poisoning that is transmitted to humans from food animals, such as pigs and chickens. Pigs and chicken are asymptomatic carriers that harbor *S*. Typhimurium mainly in the intestine. Consumption or mishandling of contaminated pork and poultry products are the primary causes of gastroenteritis in human [1, 2]. Therefore, reduction of the intestinal bacterial load and fecal excretion of the bacteria can be considered to reduce the prevalence of infection in swine and its associated risk in humans.

Despite modern antibiotic treatment, *S*. Typhimurium-related morbidity and mortality remains unacceptably high. A significant proportion of *Salmonella* isolates from humans and various species of animals, including swine, are multidrug-resistant and pose a major public health concern [3]. *S*. Typhimurium is likely to acquire and retain anti-microbial resistance genes following exposure to anti-microbial agents in animals [4]. In addition, the reluctance of the pharmaceutical industry to develop new antibiotics has further intensified the problem. Consequently, there has been considerable interest among researchers and clinicians in the use of a combination of antibiotics with natural compounds, as an alternative method to control pathogenic microorganisms. A combination of antibiotics with natural compounds are suggested to enhance the spectrum of activity, reduce the emergence of resistance, and provide synergistic effects [5–7].

Tylosin (Ty) is a macrolide antibiotic, which inhibits protein synthesis in Gram-positive pathogens and *Mycoplasma* species. In veterinary medicine, it is used primarily to control respiratory diseases in swine and other farm animals [8]. The activity of Ty is limited against Gram-negative bacteria, especially those of the Enterobacteriaceae, because of reduced penetration of the outer membrane. Although it has been banned from most of the European countries, in other parts of the world, sub-therapeutic levels of antibiotics, including Ty, are commonly used in livestock, especially in pigs, for growth promotion [9].

*Nymphaea tetragona* Georgi (family: Nymphaceae; English name: Water Lily; Korean name: Su-ryeon) is widely distributed in Asia, Europe and North America [10, 11]. In Asian traditional medicine, various parts of the plant are commonly applied to treat symptoms that are directly associated with salmonellosis such as diarrhea, dysentery, enteritis and fever [12, 13]. The authors demonstrated that methanol extract of *N*. *tetragona* inhibited the quorum sensing (QS) abilities of *Chromobacterium violaceum* and *Pseudomonas aeruginosa* [14]. Interestingly, our study also confirmed a synergistic interaction between the ethyl acetate fraction of *N*. *tetragona* (EFNTE) and Ty against *S*. Typhimurium. The major chemical compounds detected in EFNTE were methyl gallate (MG = 70.44%) and 1,2,3-benzenetriol or pyrogallol (PG = 20.61%) [15].

Therefore, the authors hypothesized the existence of synergy between Ty and the active compounds present in EFNTE against *S*. Typhimurium. Given that one or both of these compounds might be responsible for the activity of EFNTE, a screening test (minimum inhibitory concentration [MIC]- and fractional inhibitory concentration index [FICI]-based) was conducted on the anti-bacterial activities of the combination of MG with Ty (MT), and the combination of Ty with the active compound PG against *S*. Typhimurium (ATCC 14028). The result demonstrated that only MT exhibited synergism against *S*. Typhimurium.

Earlier studies on MG have shown its activity against a wide range of bacteria, including *Salmonella* when applied alone and in combination with antibiotics [16–18]. In addition, Acharyya et al. [19] and Sánchez et al. [20] demonstrated the membrane-damaging activities of MG on multidrug-resistant *Shigella* spp and *Vibrio cholerae*, respectively. The above studies examined the anti-bacterial activity of MG alone and in combination with antibiotics that are approved for the use in at least Gram-negative bacteria. However, in this study, Ty that is not a drug of choice for treatment of Gram-negative bacteria, was combined with MG as described above (MT) to evaluate the *in vitro* activity against *S*. Typhimurium. Additionally, unlike the previous studies, the effects of sub-inhibitory concentrations of MT on the interaction between *S*. Typhimurium and the host cell, as well as on the indirect host responses, were characterized.

## Materials and methods

### Chemicals and reagents

Unless specified, the chemicals and reagents used in the current study were purchased from Sigma (St. Louis, MO, USA). Stock preparations of 30 mg/mL of MG, PG and Ty were prepared in 50% ethanol and diluted in the appropriate medium (sterile distilled water and bacterial or cell culture medium). The proportion of ethanol in the final diluent never exceeded 0.5% (v/v).

### *Salmonella* strains and culture conditions

The study was conducted using *S*. Typhimurium (LVPP-STI15) from pigs, a strain that has been described and used in our previous study [21], and *S*. Typhimurium (ATCC 14028). *S*. Typhimurium was routinely cultured in Luria–Bertani (LB) agar (Difco, Sparks, MD, USA). Prior to assays, it was grown overnight in LB broth at 37°C.

### Cell culture

Human colorectal cancer (Caco-2) and RAW 264.7 macrophage cells were obtained from the Korean Cell Line Bank (Seoul, Korea). Caco-2 cells were maintained in minimum essential medium (MEM; Gibco, Grand Island, NY, USA) supplemented with 1% penicillin–streptomycin, 1% non-essential amino acids and 20% fetal bovine serum (FBS). RAW 264.7 macrophages were grown in Roswell Park Memorial Institute (RPMI) medium, containing 1% penicillin–streptomycin and 10% FBS. Cells were grown at 37°C in 5% CO_2_, and the medium was changed every other day.

### MIC and minimum bactericidal concentration (MBC)

The MICs of Ty, MG and PG against *S*. Typhimurium strains were determined using the broth microdilution method [22], with an inoculum of approximately 1×10^5^ colony-forming units per milliliter (CFU/mL) in Mueller–Hinton broth (MHB) and antibiotic-free cell culture medium. MIC was determined as the lowest concentration of the agent that inhibits visible growth, which appeared as non-turbid as judged by the naked eye after incubation at 37°C for 24 h. Aliquots from wells not showing visible growth were plated on LB agar medium. The lowest dilution concentrations that killed the bacterial inoculum after a further incubation at 37°C for 24 h were considered as the MBCs. MICs and MBCs were determined in triplicate in five independent experiments.

### Checkerboard assay

The anti-bacterial effects and interactions between Ty and both compounds (MG and PG) were assessed by the checkerboard method. Serial dilutions of MG and PG (range, ^1^/_32_× MIC–4× MIC) were diluted on separate plates and mixed with a similar MIC fold range of Ty. *S*. Typhimurium (1×10^5^ CFU/mL) was inoculated into the mixed solutions and incubated at 37°C for 24 h. Control wells were free of *S*. Typhimurium or the test compounds. The assay was performed three times in triplicate. Afterwards, the FICI was determined, as previously described (FICI ≤ 0.5, synergy; 0.5 < FICI ≤ 1.0, additivity; 1 < FICI ≤ 4.1–4.0, indifference; FICI > 4.0, antagonism) [23].

### *In vitro* time–kill assay

Based on the results of the checkerboard assay, the concentration of the combination that caused synergism (1× MT: 128 μg/mL MG and 256 μg/mL Ty), and two more concentrations combinations below the 1× MT (^1^/_4_× MT and ^1^/_2_× MT), with their respective controls, were assessed using the time–kill assay. *S*. Typhimurium (1×10^5^ CFU/mL) in MHB was incubated at 37°C with MG (32, 64, and 128 μg/mL), Ty (64, 128 and 256 μg/mL) and MT (^1^/_4_×, ^1^/_2_× and 1×) in 10 mL MHB. After 0, 1, 2, 4, 8, 12 and 24 h from the time of incubation, 100 μL was removed, and serial 10-fold dilutions were made in agar saline (0.1% agar in saline solution; Difco, Sparks, MD, USA). Then, 20 μL of the dilutions were spread-plated on LB agar plates, and the CFUs were determined after incubation at 37°C for 24 h.

### Bacterial membrane integrity

Membrane integrity was determined by using the LIVE/DEAD BacLight kit (Molecular Probes, Eugene, OR, USA) with a slight modification of the manufacturer’s instructions. *S*. Typhimurium (10^6^ CFU/mL) was treated as described in the time–kill assay, for 4 h. Three microliters of SYTO9 and a propidium iodide (PI) mixture (1:1) were added to 1 mL of pre-treated bacterial cultures and incubated in the dark for 15 min. Samples were then visualized using a Zeiss confocal microscope (LSM700, Carl-Zeiss, Jena, Germany) at excitation/emission wavelengths of 480/500 and 490/635 nm for SYTO9 and PI stain, respectively.

### Scanning electron microscope (SEM) analysis

The effect of MT on bacterial membrane integrity was further examined using an SEM, with a slight modification of the method described by [24]. *S*. Typhimurium (10^7^ CFU/mL) was treated as described in the time–kill assay at 37°C for 6 h. Bacteria were centrifuged (10,000 *g* for 15 min) and washed twice with 0.9% saline solution, re-suspended in 2.5% glutaraldehyde and maintained at –4°C for 12 h. The resultant cells were centrifuged (10,000 *g* for 8 min) and dehydrated in ethanol (30, 50, 70, 80, 90 and 100%) for 10 min. The samples were further dried and examined under an SEM (SU8200 Hitachi, Tokyo, Japan).

### Determination of membrane potential (MP)

The change in the differences in electric potential that exists across the intact bacterial cell membrane (i.e., the MP) following MT treatment was determined using the BacLight bacterial MP kit (Molecular Probes). *S*. Typhimurium (10^7^ CFU/mL) was diluted in phosphate-buffered saline (PBS) and treated similarly to the time–kill assay, for 2 h. Afterwards, 10 μL (3 mM) of 3,3-diethyloxacarbocyanine iodide was added to 1 mL of the bacterial suspension and mixed well. Ten microliters of 500 μM carbonyl cyanide 3-chlorophenylhydrazone was added into the control tube while an equal volume of PBS was added to the non-treated control. Following incubation at room temperature for 15 min, the samples were analyzed using flow cytometry (BD Biosciences, San Jose, CA, USA) at excitation and emission filters of 488 and 515 nm, respectively. The MP was determined based on the ratio of the red-to-green fluorescence intensity.

### Biofilm inhibition and dispersion assay

The effect of sub-inhibitory concentrations of MG (2–128 μg/mL), Ty (2–256 μg/mL) and MT (^1^/_128_× MT–^1^/_4_× MT) on *S*. Typhimurium biofilm was evaluated using a minor modification of a previous method [25]. Overnight cultures of *S*. Typhimurium grown in tryptic soy broth were normalized to an OD_600_ of 0.8. Two hundred microliters of a 1:100 dilution (in tryptic soy broth) of the normalized cultures was dispensed in 96-well plates, treated with different concentrations of MG, Ty and MT, and incubated at 30°C under static condition. Control plates were not treated with any of the test substances. Following a 24-h incubation, the planktonic bacteria growth was confirmed using a microplate reader (optical density at 600 nm). The plates were then washed with sterile double-distilled water, fixed at 60°C for 1 h and stained with 0.1% crystal violet (CV) in water. After incubation at room temperature for 15 min, the CV was solubilized using 30% acetic acid in water, and the absorbance was determined at 550 nm against the blank (30% acetic acid in water). The concentrations of MG, Ty and MT that resulted in 50% inhibition of the maximum biofilm formation (EC_50_) were determined. Furthermore, the impact of MT on biofilm dispersion was measured in cells treated after 24 h of biofilm formation and incubated further for 24 h.

### Cytotoxicity assay

The cytotoxic effects of MG, Ty and MT on Caco-2 and RAW 264.7 cells were determined using the 3-(4,5-dimethyl-2-thiazolyl)-2,5-diphenyl-2H-tetrazolium bromide (MTT) assay described by [26]. The absorbance was determined at 570 nm using a VersaMax microplate reader (Molecular Devices, Sunnyvale, CA, USA), and the percentage viability of cells was determined as follows.

Percentage viability = (OD value of treated cells/OD value of control cells) ×100

where OD is the optical density.

### Adhesion and invasion assay

The gentamicin protection assay [27] was conducted to evaluate the impacts of different concentrations of MG, Ty and MT on the adhesion and invasion capacity of *S*. Typhimurium to Caco-2 cells. Cells (10^5^/mL) were grown in 24-well plates in antibiotic-free medium. Cells were treated with 900 μL of MG (8, 16 and 32 μg/mL), Ty (16, 32 and 64 μg/mL), ^1^/_16_× MT (8 μg/mL MG and 16 μg/mL Ty), ^1^/_8_× MT (16 μg/mL MG and 32 μg/mL Ty) and ^1^/_4_× MT (32 μg/mL MG and 64 μg/mL Ty) for 45 min. Then, *S*. Typhimurium was recovered by centrifugation at 10,000 *g* for 15 min, washed twice with 1× PBS and re-suspended in antibiotic-free MEM (10^7^ CFU/mL). One hundred microliters of bacterial suspensions (MOI = 10:1) were added to each wells, centrifuged at 1000 *g* for 2 min and incubated at 37°C for 45 min. The supernatant was discarded, and the cells were washed with 1× PBS to remove non-adhered bacteria. Cells were disrupted with 1% Triton X-100. The lysates were serially diluted using agar saline and plated on LB agar. The total number of adhering bacteria (CFU/mL) was determined after incubation at 37°C for 16–18 h.

For the invasion assay, treated and infected cells were incubated at 37°C for 60 min. The supernatant was discarded, and non-adhered bacteria were removed by washing three times with 1× PBS, followed by addition of 300 μL gentamicin (100 μg/mL) in MEM and further incubation for 60 min to kill extracellular bacteria. Cell lysis and determination of the number of invading bacteria were performed similarly to the procedure mentioned above in the adhesion assay.

### Intracellular survival assay

Survival of *S*. Typhimurium in macrophages pre-treated with MG, Ty and MT was determined by slight modifications of the methods described by Lu et al. [28]. Cells (10^5^/mL) grown without antibiotics in 24-well plates were treated and infected, as described above in the invasion assay. After incubation with gentamicin (100 μg/mL) at 37°C for 60 min, cells were washed with 1× PBS, and 25 μg/mL gentamicin in RPMI was added for the remaining incubation time. At 2, 4 and 8 h post-incubation, cells were lysed, and the total number of surviving bacteria was determined as described above.

### Detection of cytokine expression

Infection of intestinal epithelial cells with *S*. Typhimurium up-regulates the expression of many cytokine genes [29]. The effects of MG, Ty and MT on *S*. Typhimurium (ATCC 14028)-induced cytokine (tumor necrosis factor alpha [TNFα], and interleukin [IL]-1β, IL-6, IL-8 and IL-10) gene expression in Caco-2 cells was evaluated using quantitative real-time reverse transcription PCR (qRT–PCR). For this purpose, cells (10^5^/mL) were treated and infected, as described above in the adhesion assay. After 8-h incubation, total RNA was extracted using TRIzol (Ambion Life Technologies, Carlsbad, CA, USA). The concentration and purity of RNA were determined using Nanophotometer (Implen GmbH, Munich, Germany). An *A*_260_/*A*_280_ ratio of 1.8 to 2.0 was considered as good quality RNA and a total of 1μg was taken for cDNA synthesis. The cDNA was amplified, and qRT–PCR was performed in a CFX96 Touch real-time PCR detection system (Bio-Rad, Hercules, CA, USA) using IQ SYBR Green Supermix (Bio-Rad Laboratories [Singapore] Pte. Ltd.). Three hundred Nano molars of both the forward and reverse primers were used in 20 μL reaction mixture. The reaction conditions included denaturation at 95°C for 3 min, 40 cycles of amplification at 94°C for 10 s, 60°C for 30 s and melting from 65 to 90°C. The housekeeping genes β-actin and GADPH were used to normalize gene expression. The fold changes of cytokines gene expression were analysed using the 2^-ΔΔCT^ method and the statistical difference were determined using the Student’s t-test in GraphPad Prism 6 (GraphPad Software, Inc., San Diego, CA, USA). The sequences of all primers used in this experiment are listed in S1 Table.

### Data analysis

Data were analyzed using GraphPad Prism 6 (GraphPad Software, Inc., San Diego, CA, USA). One-way and two-way analyses of variance (ANOVA) were conducted to compare the mean values among treatment groups. *P* < 0.05 was considered statistically significant.

## Results

### MIC and MBC

The MICs of MG, Ty and PG against the two *S*. Typhimurium strains were 512, 1024 and 128 μg/mL, respectively. The MBCs of MG and PG were twice the MIC values in both strains; however, the MBC of Ty exceeded 4 mg/mL. Similar MIC values were obtained for both of the tested agents in RPMI and MEM.

### Checkerboard assay

MT was effective, as confirmed by the reduced MICs of 128 and 256 μg/mL for MG and Ty, respectively, for both strains. The FICI was calculated to be 0.5, indicating synergism between Ty and MG. Furthermore, an additive effect (FICI = 0.56) was found between Ty and PG, in which the MIC values were reduced to 64 μg/mL. Therefore, MT was chosen for subsequent experiments.

### Time–kill assay

**Fig 1:**
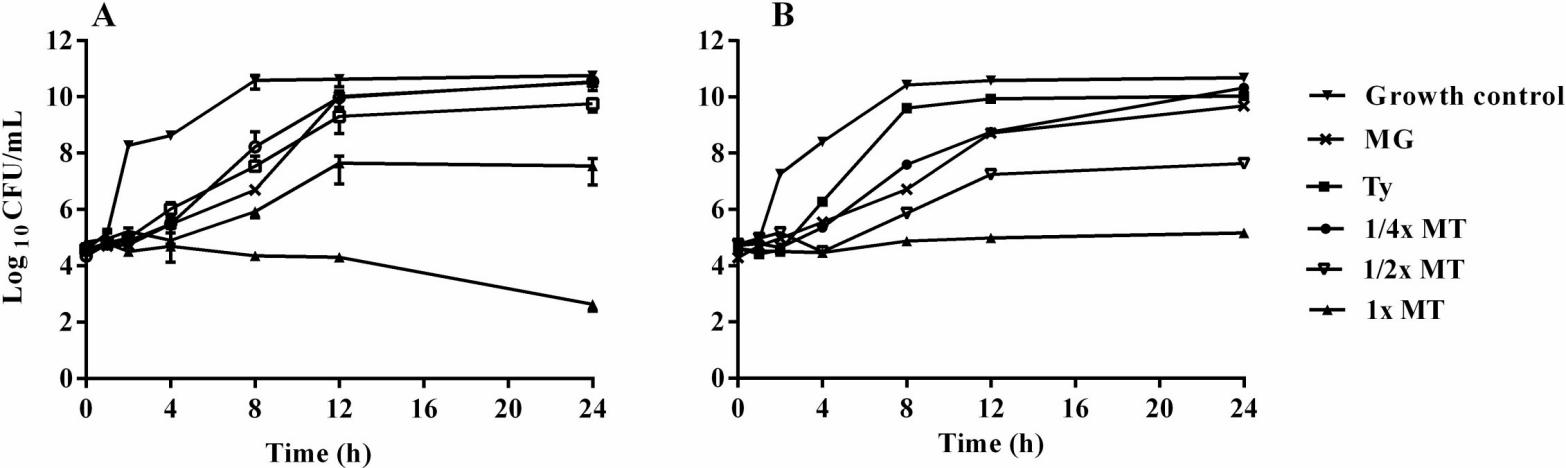
**Time-kill curves of *S*. Typhimurium ATCC 14028 (A) and *S*. Typhimurium LVPP-STI15 (B) treated with methyl gallate (MG), tylosin (Ty) and various concentrations of their combination (MT).** Plotted values represent mean ± SEM of at least six independent experiments. Growth control: non-treated *S*. Typhimurium. MG = 128 μg/mL; Ty = 256 μg/mL; ¼× MT = 32 μg/mL MG and 64 μg/mL Ty; ½× MT = 64 μg/mL MG and 128 μg/mL Ty; and 1× MT = 128 μg/mL MG and 256 μg/mL Ty. Since there was no concentration-dependent difference in the killing activity of MG (32, 64 and 128 μg/mL) and Ty (64, 128 and 256 μg/mL), only the growth curves of the bacteria treated with the maximum concentrations of each agent are displayed.

To confirm the results of the checkerboard assay, the *in vitro* time–kill activities of MT were performed for both strains (Fig 1). At 1× MT, a synergistic effect occurred that caused more than 2 log_10_ CFU/mL reduction compared with Ty and MG alone. Conversely, the growth curves of *S*. Typhimurium treated with 64 and 128 μg/mL Ty were almost the same as those treated with 256 μg/mL Ty. Likewise, the differences in the growth curves of *S*. Typhimurium treated with 32, 64 and 128 μg/mL MG were not significant.

### Bacterial membrane integrity

**Fig 2:**
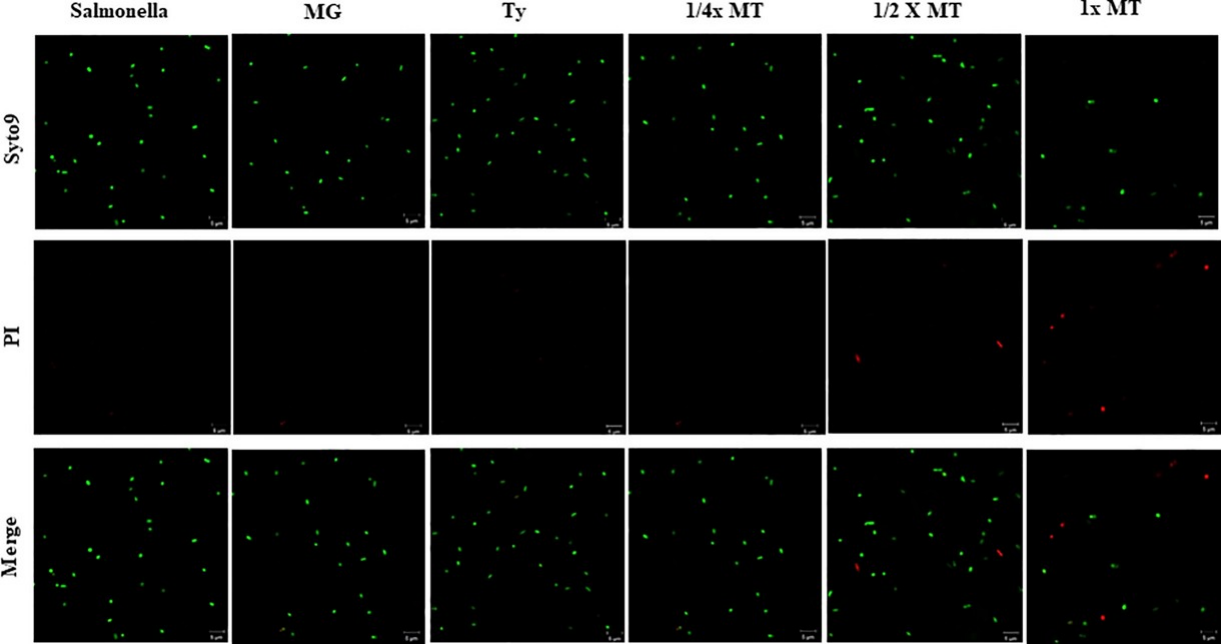
Confocal laser scanning microscopy analysis of the membrane integrity of *S*. Typhimurium (ATCC 14028) treated with methyl gallate (MG), tylosin (Ty) and various concentrations of their combination (MT). SYTO9/PI staining was applied to differentiate cells with intact membranes (green) from those with damaged membranes (red). *Salmonella*: non-treated *S*. Typhimurium. MG = 128 μg/mL; Ty = 256 μg/mL; ¼× MT = 32 μg/mL MG and 64 μg/mL Ty; ½× MT = 64 μg/mL MG and 128 μg/mL Ty; and 1× MT = 128 μg/mL MG and 256 μg/mL Ty.

The confocal microscopic images of SYTO9/PI-stained *S*. Typhimurium exposed to MG, Ty and MT for 4 h are illustrated in Fig 2. *S*. Typhimurium with a damaged membrane (red fluorescent stain) were observed after 4-h exposure to ^1^/_2_× MT (5.8 ± 1.2%) and 1× MT (21.2 ± 4.8%). In contrast, those grown in the presence of MG, Ty and 1/4× MT (green fluorescent stain) indicated that the cell membrane was intact.

### Field emission scanning electron microscope (SEM) analysis

**Fig 3:**
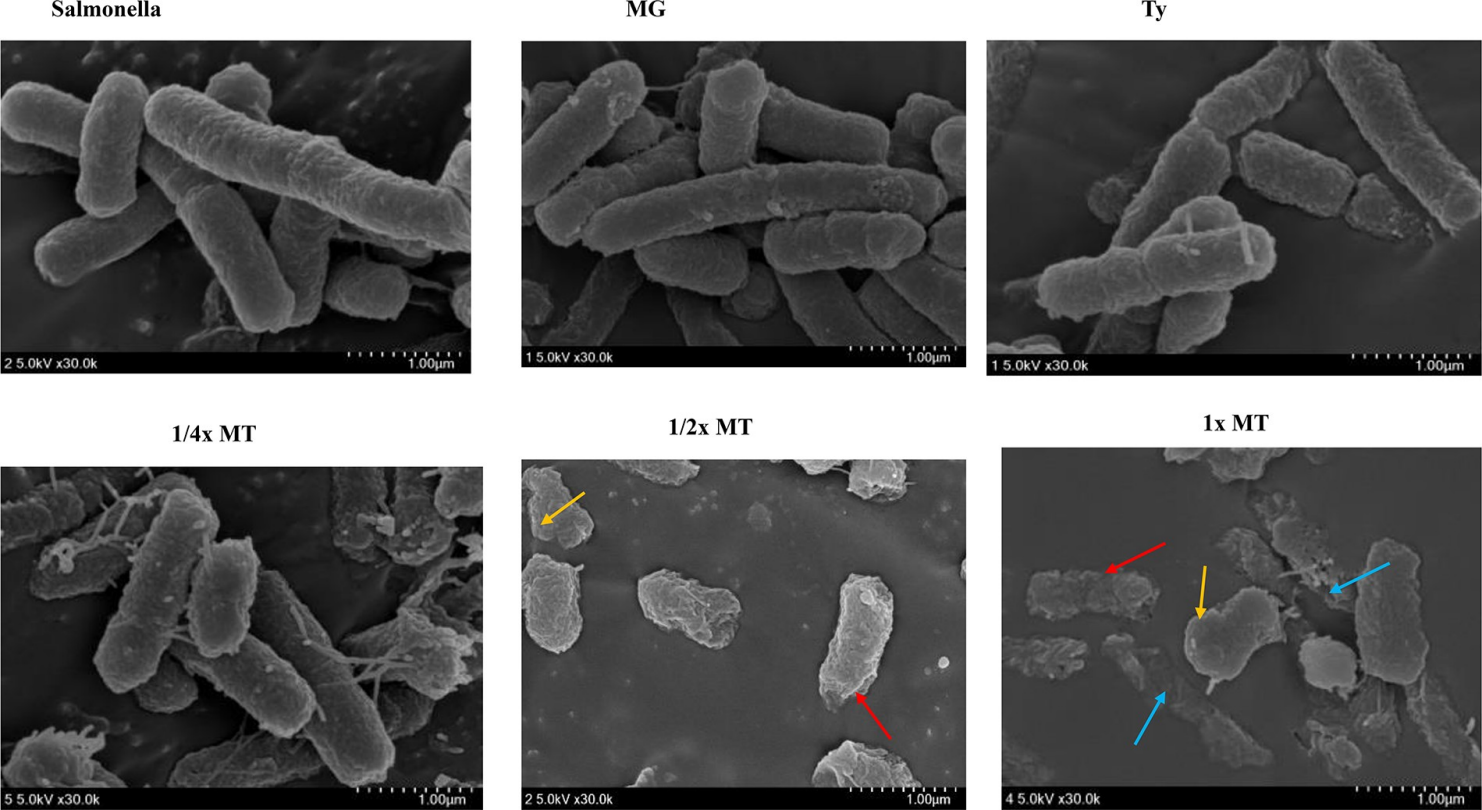
Scanning electron microscopic analysis of *S*. Typhimurium (ATCC 14028) treated with methyl gallate (MG), tylosin (Ty) and various concentrations of their combination (MT). Untreated control and *Salmonella* treated with MG, Ty and ¼× MT demonstrated intact morphology. *S*. Typhimurium treated with 1x MT and ½x MT showed altered morphology and leakage of cellular contents. *Salmonella*: non-treated *S*. Typhimurium. MG = 128 μg/mL; Ty = 256 μg/mL; ¼× MT = 32 μg/mL MG and 64 μg/mL Ty; ½× MT = 64 μg/mL MG and 128 μg/mL Ty; and 1× MT = 128 μg/mL MG and 256 μg/mL Ty.

For both strains of *S*. Typhimurium, treatment with ^1^/_2_× MT and 1× MT caused a remarkable modification in morphology, such as rough surface (red arrow), membrane blebbing (yellow arrow) and leakage of cellular contents (blue arrow) (Fig 3). Marked membrane disintegration with leakage of cellular contents was evident in the 1× MT-treated *S*. Typhimurium compared with the non-treated control, as well as those treated with MG and Ty alone.

### Resting MP

**Fig 4:**
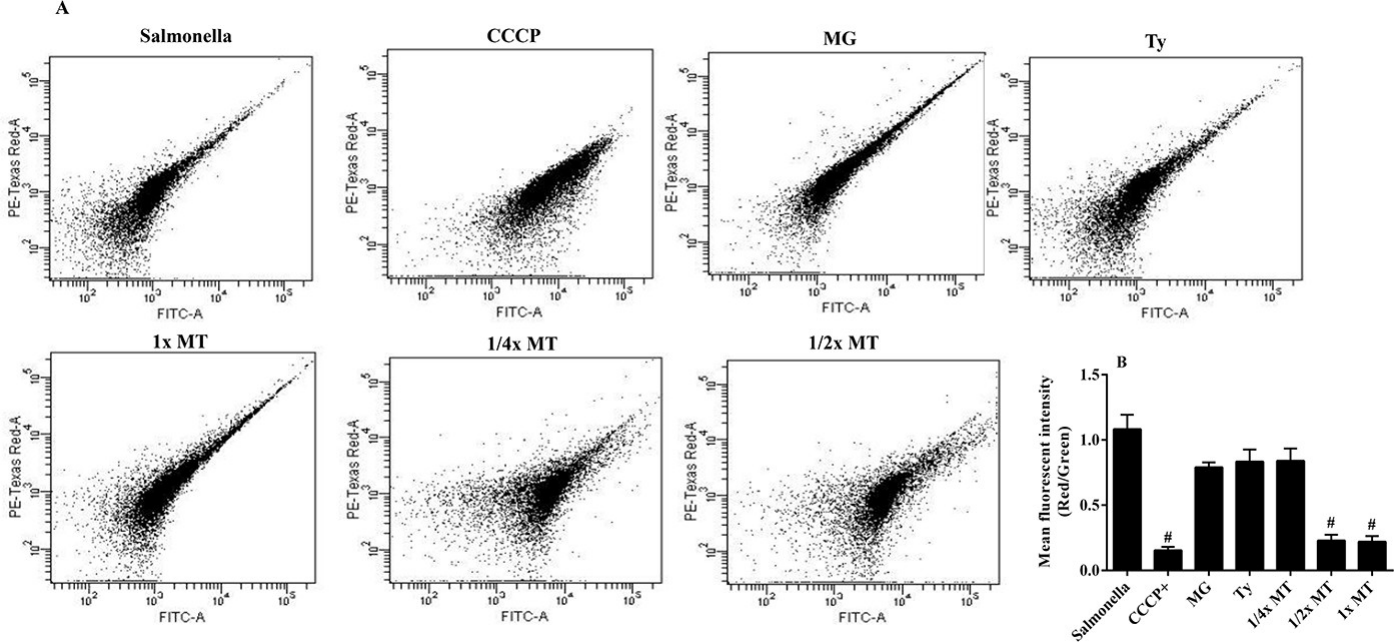
Effect of treatment with methyl gallate (MG), tylosin (Ty) and various concentrations of their combination (MT) on the membrane potential of *S*. Typhimurium (ATCC 14028). The flow cytometry depicts cells exhibiting both fluorescence intensities (**A**). The membrane potential (red-to-green fluorescent ratio) of *S*. Typhimurium treated with various agents, including the well-known depolarizing agent, carbonyl cyanide 3-chlorophenylhydrazone (CCCP) (**B**). ^#^*P* < 0.001 compared to both MG and Ty. *Salmonella*: non-treated *S*. Typhimurium. MG = 128 μg/mL; Ty = 256 μg/mL; ¼× MT = 32 μg/mL MG and 64 μg/mL Ty; ½× MT = 64 μg/mL MG and 128 μg/mL Ty; and 1× MT = 128 μg/mL MG and 256 μg/mL Ty.

The MP of *S*. Typhimurium (ATCC 14028) that had been incubated with different concentrations of MT for 2 h was determined by measuring the ratio of red-to-green fluorescence intensity (Fig 4). The ratio was decreased to 0.20 in *S*. Typhimurium treated with ^1^/_2_× MT and 1× MT. The value was significantly (*P* < 0.001) lower when compared with fluorescence ratios of 0.80, 0.83 and 1.10 in MG-treated, Ty-treated and non-treated *S*. Typhimurium, respectively. However, treatment with ^1^/_4_× MT did not produce a significant change in the fluorescence ratio (*P* > 0.05).

### Biofilm formation

**Fig 5:**
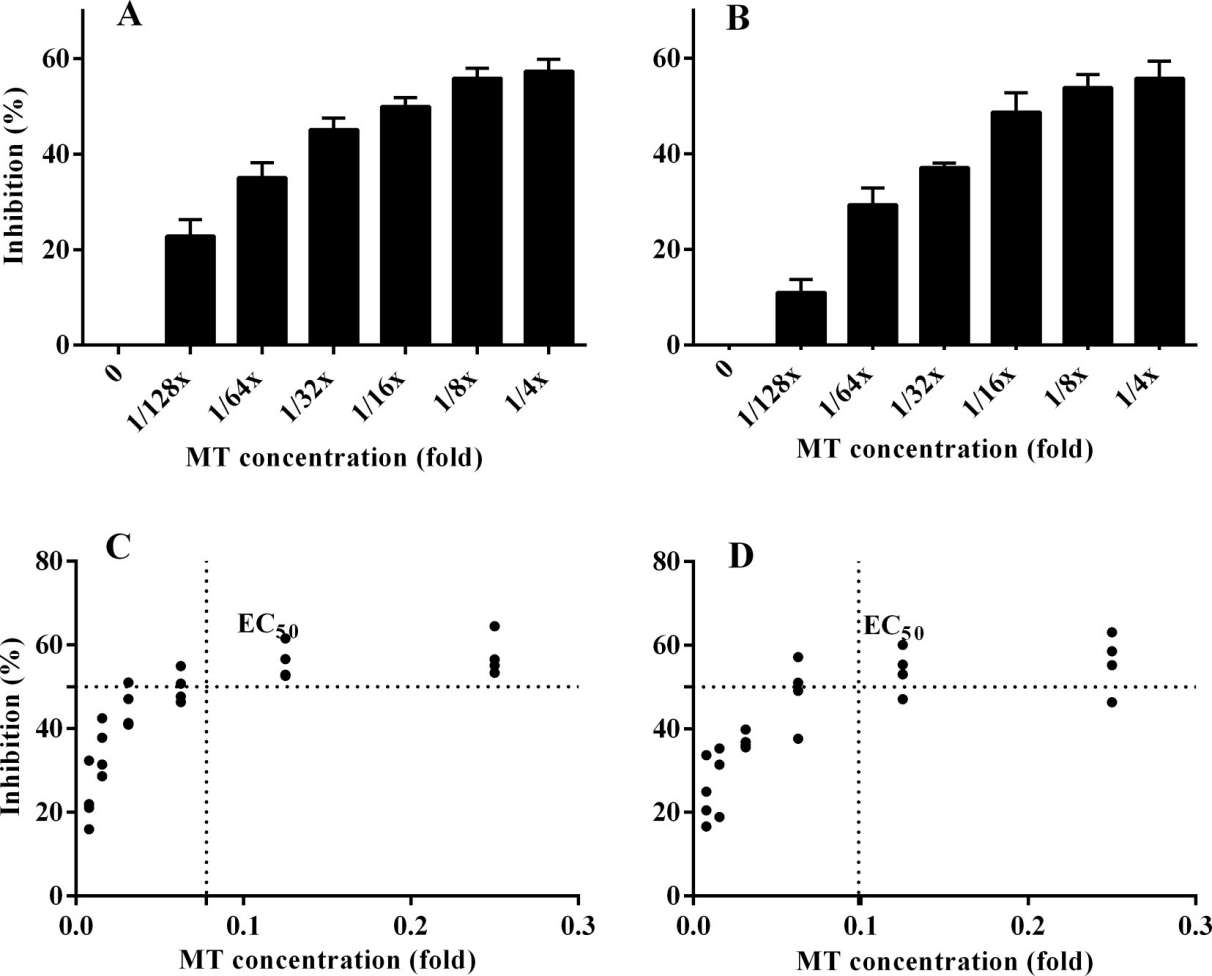
Impacts of treatment on *S*. Typhimurium biofilms. The figures demonstrated a concentration-dependent inhibition of *S*. Typhimurium (ATCC 14028) (A) and LVPP-STI15 (B) biofilm formation in the presence of a combination of methyl gallate (MG) and tylosin (Ty) (MT) with an initial concentration of ¼× MT (32 μg/mL MG and 64 μg/mL Ty). The EC_50_ of MT in *S*. Typhimurium (ATCC 14028) (C) and LVPP-STI15 (D) biofilms were calculated by considering 0% and 100% as minimal and maximal biofilm inhibition, respectively. Values represent mean ± SEM of four independent experiments.

The study demonstrated an insignificant difference (*P* > 0.05) in planktonic bacterial growth (mean OD_600_) in the presence and absence of sub-inhibitory concentrations of MG, Ty and MT. Particularly, MT reduced *S*. Typhimurium biofilm formation in a concentration-dependent manner. Biofilm formation by *S*. Typhimurium (ATCC 14028) treated with ^1^/_8_× MT and ^1^/_4_× MT was decreased by 57.3 and 55.9%, respectively (Fig 5). The EC_50_ of MT was calculated for both strains of *S*. Typhimurium, considering 0 and 100% as the minimal and maximal biofilm inhibition, respectively. Accordingly, ^1^/_10_× MT (a combination of 12.8 μg/mL MG and 25.6 μg/mL Ty) could cause half of the maximum inhibition (EC_50_) of *S*. Typhimurium (LVPP-STI15) biofilms while the EC_50_ value for *S*. Typhimurium ATCC 14028 biofilms was ^1^/_12.5_× MT (a combination of 10.24 μg/mL MG and 20.5 μg/mL Ty). Conversely, the maximum biofilm inhibitions caused by MG and Ty were 6.86 and 6.48%, respectively (S1 Fig). There was no significant difference (*P* > 0.05) in the biofilm inhibition ability of MT between the two strains. Besides, neither the combination nor each agent dispersed the pre-formed biofilms of both strains.

### Cytotoxicity assay

**Fig 6:**
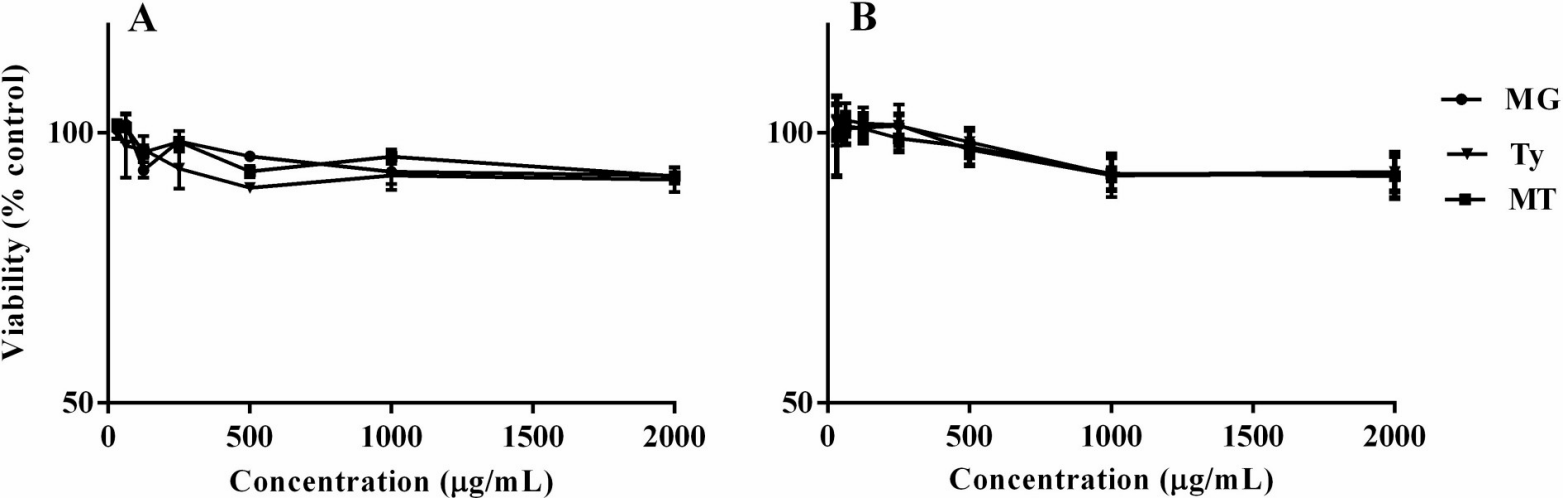
**The viability of RAW 264.7 macrophages (A) and Caco-2 epithelial cells (B) exposed to different concentrations of methyl gallate (MG), tylosin (Ty) and their combination (MT).** Percentage viability was calculated relative to the non-treated control cells. The initial 2000 μg/mL MT concentration represents a combination of 2000 μg/mL MG and 2000 μg/mL Ty. Values represent mean ± SEM of three independent experiments.

The effects of MG, Ty and MT on the viability of Caco-2 epithelial cells and RAW 264.7 macrophages are shown in Fig 6. Treatment of cells with MG and Ty individually, at initial concentrations of 2 mg/mL, did not produce a significant toxic effect. Likewise, greater than 90% of the cells were viable following 24 h of exposure to MT containing initial concentrations of 2 mg/mL MG and 2 mg/mL Ty.

### Adhesion and invasion assay in Caco-2 cells

**Fig 7:**
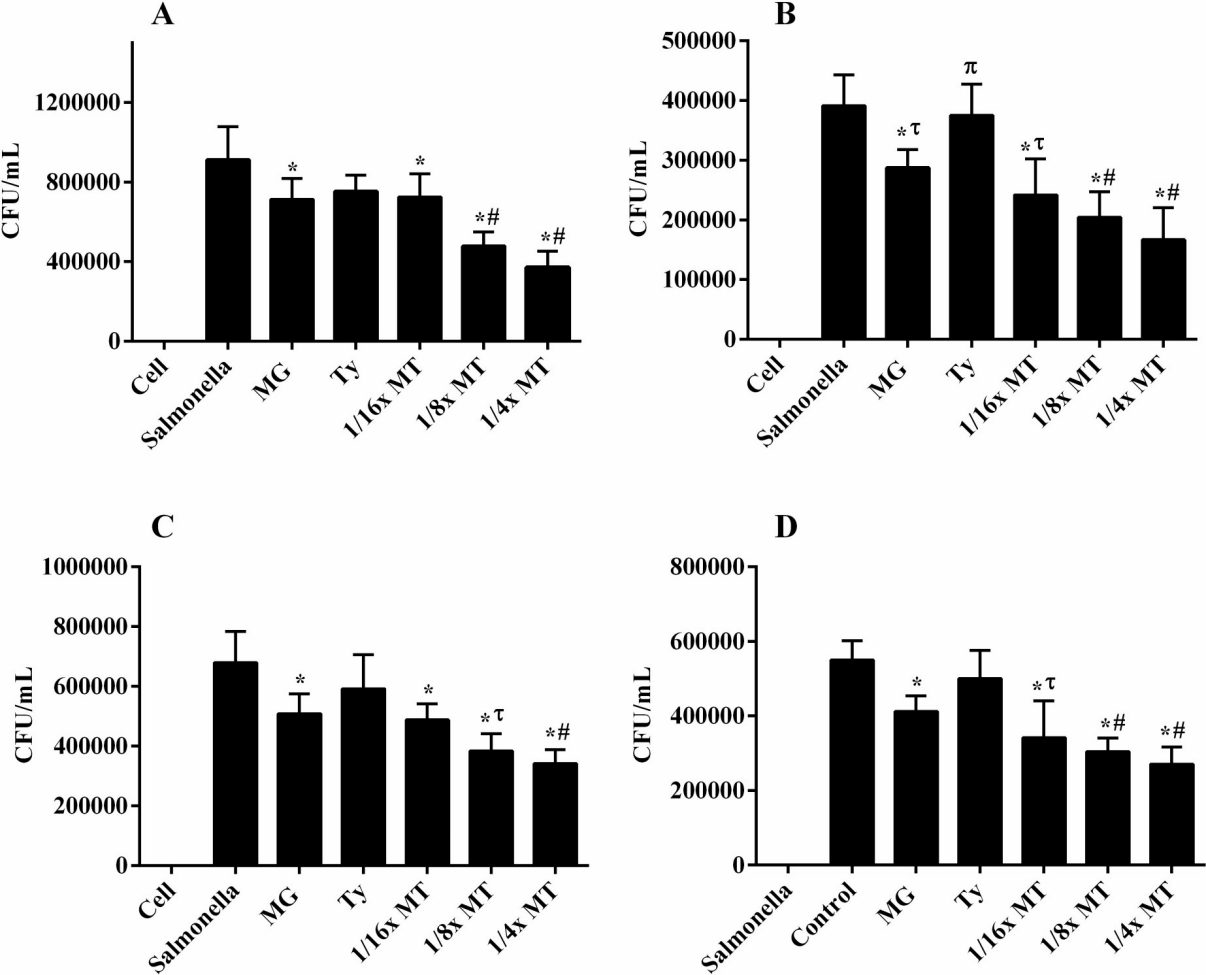
**Effects of methyl gallate (MG), tylosin (Ty) and various concentrations of their combination (MT) on the adhesion and invasion of *S*. Typhimurium ATCC 14028 (A and C) and *S*. Typhimurium LVPP-STI15 (B and D) to CaCo-2 intestinal epithelial cells.** Cell: non-infected and non-treated control. *Salmonella*: *S*. Typhimurium infected, but non-treated cells. Values represent mean ± SEM of six independent experiments. MG = 32 μg/mL; Ty = 64 μg/mL; ^1^/_16_× MT = 8 μg/mL MG and 16 μg/mL Ty; ^1^/_8_× MT = 16 μg/mL MG and 32 μg/mL Ty; and ¼× MT = 32 μg/mL MG and 64 μg/mL Ty. *P < 0.05 compared to infected, but non-treated cells; ^τ^*P* < 0.05 compared to Ty; ^π^*P* < 0.05 compared to MG; and ^#^*P* < 0.05 compared to both MG and Ty. The effects of MG (8, 16 and 32 μg/mL) and Ty (16, 32 and 64 μg/mL) were not concentration-dependent.

The effects of sub-inhibitory concentrations of MG, Ty and MT on the adhesion and invasion of *S*. Typhimurium to Caco-2 epithelial cells are demonstrated in Fig 7. Adhesion of *S*. Typhimurium LVPP-STI15 and ATCC 14028 to Caco-2 cells was strongly decreased in cells pre-treated with 1/_16_× MT (81.3 and 70.4%, respectively) (*P* < 0.05), ^1^/_8_× MT (53.7 and 55.8%, respectively) (*P* < 0.0001) and ^1^/_4_× MT (38.5 and 48.7%, respectively) (*P* < 0.001) relative to the infected and non-treated cells. The counts of adhered *S*. Typhimurium (ATCC 14028) in cells pre-treated with ^1^/_8_× MT and ^1^/_4_× MT were significantly lower relative to those pre-treated with MG (73.6%) (*P* < 0.01) and Ty (86.1%) (*P* < 0.001) alone. Moreover, the invasion of *S*. Typhimurium strains LVPP-STI15 and ATCC 14028 to Caco-2 cells was reduced in a concentration-dependent manner in cells pre-treated with ^1^/_16_× MT (57.1 and 60.2%, respectively), ^1^/_8_× MT (53.7 and 49.4%, respectively) and ^1^/_4_× MT (50.0 and 40.4%, respectively) when compared with the infected and non-treated cells (*P* < 0.0001). The counts of invaded bacteria in cells treated with ^1^/_4_× MT and ^1^/_8_× MT was significantly reduced with respect to those of MG (*P* < 0.05) and Ty (*P* < 0.001) alone. Interestingly, the total counts of adhered (*P* < 0.05) and invaded (*P* < 0.01) bacteria reduced significantly in the presence of MG alone in comparison to infected and untreated cells, but to a lesser extent compared to MT.

### Effects of MT on intracellular bacterial survival

**Fig 8:**
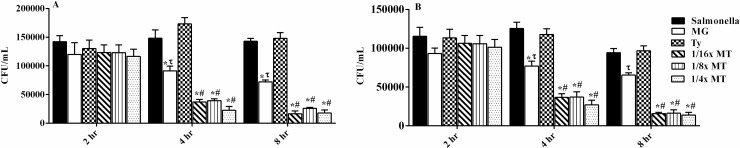
**Efficacy of methyl gallate (MG), tylosin (Ty) and various concentrations of their combination (MT) on the intracellular killing of *S*. Typhimurium ATCC 14028 (A) and *S*. Typhimurium LVPP-STI15 (B) in macrophages at 2, 4 and 8 h post-treatment and infection.** Cell: non-infected and non-treated control. *Salmonella*: *S*. Typhimurium infected, but non-treated cells. Values represent mean ± SEM of six independent experiments. MG = 32 μg/mL; Ty = 64 μg/mL; ^1^/_16_× MT = 8 μg/mL MG and 16 μg/mL Ty; ^1^/_8_× MT = 16 μg/mL MG and 32 μg/mL Ty; and ¼× MT = 32 μg/mL MG and 64 μg/mL Ty. *P < 0.05 compared to infected, but non-treated cells; ^τ^*P* < 0.05 compared to Ty; ^π^*P* < 0.05 compared to MG; and ^#^*P* < 0.05 compared to both MG and Ty.

After incubation for 2 h, treatment did not produce a significant difference in the percentage of surviving bacteria (Fig 8). However, a marked decrease in the percentage of intracellular *S*. Typhimurium was observed after incubation with various concentrations of MT for 4 and 8 h in comparison to MG and Ty alone. The bacterial load (ATCC 14028 and LVPP-STI15) in cells incubated with ^1^/_16_× MT, ^1^/_8_× MT and ^1^/_4_× MT for 8 h was decreased significantly (*P* < 0.001) by 79.14 and 88.6%, 79.1 and 81.99%, and 82.7 and 87.75%, respectively, compared with the load in the infected and non-treated cells. Interestingly, MG significantly (*P* < 0.5) decreased the intracellular *S*. Typhimurium count from 4 h of incubation, in contrast with Ty, as well as the infected and non-treated cells.

### Cytokine expression

**Fig 9:**
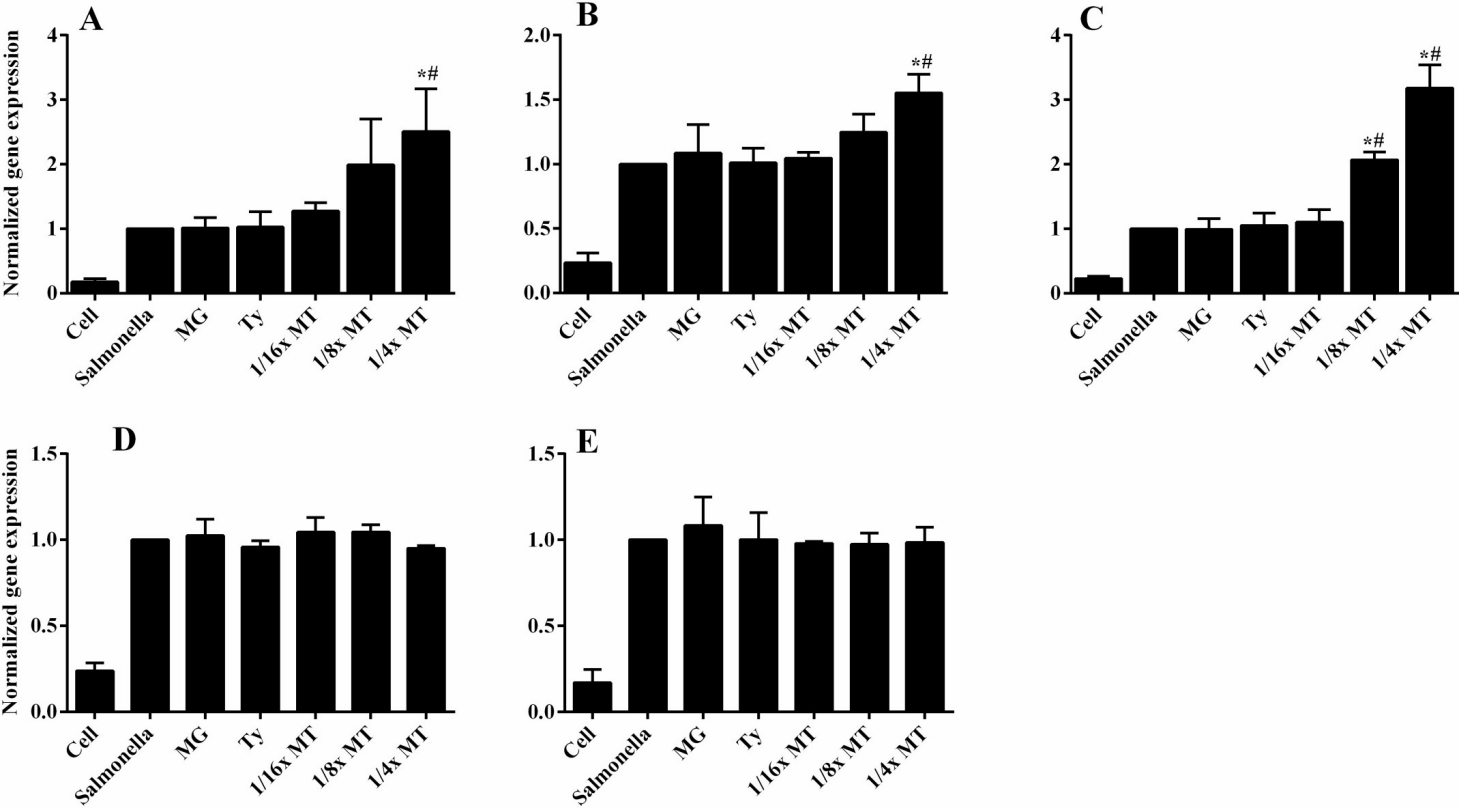
**Cytokine expression of IL-8 (A), IL-10 (B), IL-6 (C), IL-1β (D) and TNFα (E) in Caco-2 monolayers co-cultured with methyl gallate (MG), tylosin (Ty) and various concentrations of their combination (MT) during 8 h infection with *S*. Typhimurium.** Cell: non-infected and non-treated control. *Salmonella*: infected, but non-treated cells. Results mean ± SEM of three independent experiments. MG = 32 μg/mL; Ty = 64 μg/mL; ^1^/_16_× MT = 8 μg/mL MG and 16 μg/mL Ty; ^1^/_8_× MT = 16 μg/mL MG and 32 μg/mL Ty; and ¼× MT = 32 μg/mL MG and 64 μg/mL Ty. *P < 0.05 compared to infected, but non-treated cells; ^τ^*P* < 0.05 compared to Ty; ^π^*P* < 0.05 compared to MG; and ^#^*P* < 0.05 compared to both MG and Ty.

*S*. Typhimurium induced the expression of IL-6, IL-8, IL-1β, TNFα and IL-10 genes in CaCo-2 cells after 8-h infection (Fig 9). A concentration-dependent up-regulation (*P* < 0.001) in the IL-6 gene expression was detected when infected cells were incubated with ^1^/_8_× MT and ^1^/_4_× MT compared to those of MG and Ty. The expression of IL-8 gene was significantly enhanced (*P* < 0.01) in ^1^/_4_× MT-treated cells. Treatment by ¼ x MT up-regulated the gene expression of IL-10 to a very moderate level (1.5X) by comparison to IL-6 (2.5X) and IL-8 (3X). However, neither MG nor Ty modified the genes expressions of the tested cytokines in infected cells. In addition, the effect of MT on *S*. Typhimurium-induced IL-1β and TNFα gene expression was not significantly different from those of MG and Ty alone. Moreover, the results of genes expressions of cytokines normalized to GADPH housekeeping gene exhibited similar patterns as shown on supplementary file (S2 Fig).

## Discussion

The high MIC value of Ty (1024 **μ**g/mL) found in this study is not surprising because the outer membrane of Enterobacteriaceae, such as *Salmonella*, has low permeability to macrolides, including Ty, which limits the activities of these drugs [30]. The MIC of MG (512 **μ**g/mL) was comparable to the findings of Choi et al. [16] but much higher than the values (3.9–125 **μ**g/mL) reported by Choi et al. [17] and Choi et al. [18] on *Salmonella* isolates from chicken and pig. The difference of bacterial strains used between these studies could contribute to their difference in susceptibility to MG.

The FICI and the time–kill assay confirmed *in vitro* synergy since more than a 2-log-fold reduction in CFU/mL was found when MT was used against *S*. Typhimurium at concentrations lower than the MICs of the individual components. The SYTO9/PI staining and SEM analysis further supported these findings, in which bacterial death, membrane damage or disintegration and leakage of cellular contents were exhibited following MT treatment. Proteins and small molecules are believed to cause anti-bacterial action by perforating the bacterial membrane and causing leakage of cellular contents [31]. The ability of 1× MT to induce leakage of cellular contents indicates that it acts to cause bacterial membrane destabilization and perforations. This could lead to a further influx of MG and Ty across the damaged membrane to the intracellular target sites. Previous studies have confirmed the membrane-damaging activity of MG when used alone against *V*. *cholera* [20] and multidrug-resistant *Shigella* spp. [19] but at relatively higher concentrations (1× MBC–5× MBC).

Reduction in the ratio of red-to-green fluorescent of *S*. Typhimurium following SYTO9/PI staining is an indicator of an altered MP [32]. Studies on carvacrol and thymol confirmed that agents that contain free hydroxyl groups could act as a protonophore [32–34], enhancing insertion into the bacterial membrane and disrupting the physical, as well as chemical properties, of the membrane. The lipid layer becomes destabilized, resulting in an increase of passive proton flux across the membrane and contributes to alteration in the MP [34, 35]. Therefore, the free hydroxyl groups in MG could contribute to the membrane destabilization effects of MT. In addition, MT-induced loss of *S*. Typhimurium membrane integrity could also lead to leakage of protons and potassium, which results in the change in MP. Studies on various bacterial species exhibited that natural products could alter the MP and ultimately lead to cell death [33–35]. Therefore, the anti-bacterial activity of MT could involve disruption of the MP, as well as membrane disintegration.

A number of studies have demonstrated the role played by biofilms in anti-microbial resistance and persistence. *S*. Typhimurium is known to be able to produce biofilms on diverse surfaces, including the gallbladder, epithelial cells and various host tissue compartments [36, 37]. The CV staining demonstrated a concentration-dependent reduction of *S*. Typhimurium biofilm formation. The activity of MT against *S*. Typhimurium biofilms was not a function of the direct anti-bacterial activity, as it was confirmed in the time–kill and membrane integrity assays. QS-ability is suggested to play a critical role in biofilm formation. Furthermore, exopolysaccharides, such as cellulose, are one of the most important extracellular polymeric structures for biofilm formation in *S*. Typhimurium [37, 38]. Our previous studies demonstrated that sub-MIC of MG reduced exopolysaccharide production and QS in *P*. *aeruginosa* biofilms [39] and *S*. Typhimurium [40], respectively. *In vitro* studies have also indicated that macrolides alter biofilm architecture through inhibition of polysaccharide synthesis in *P*. *aeruginosa* [41]. Moreover, the anti- QS [42] and biofilm inhibitory activities of sub-MIC concentrations of macrolides [43] and MG [39] are reported in various bacterial species. Therefore, MG and Ty could act synergistically to inhibit biofilm formation in *S*. Typhimurium.

The ability to adhere, invade and survive in intestinal epithelial cells and macrophages are critical for the pathogenesis of *Salmonella*. Thus, agents that could interfere with the adherence and invasion of *S*. Typhimurium to intestinal epithelial cells play a critical role to avoid the establishment of infection [44]. In addition, the importance of motility for bacterial invasion has already been confirmed [45]. In the current study, MT treatment reduced the adhesion, invasion and intracellular survival of *S*. Typhimurium. Similar effects were also found following treatment of cells with MG, but to a lesser extent compared with MT. Low levels of macrolides, such as erythromycin, are reported to reduce the adherence of *P*. *aeruginosa* to airway epithelial cells [46]. Moreover, our recent study confirmed the inhibitory effects of MG on *S*. Typhimurium motility and invasion into host cells [40]. Therefore, the synergistic interaction between the two agents could contribute to the significant reduction in the adhesion, invasion and survival of *S*. Typhimurium to the host cells.

The balance between the host response and the bacterial virulence mechanisms determines the outcome of infection. The *S*. Typhimurium-induced release of cytokines by intestinal epithelial cells are critical to initiate and orchestrate the inflammatory events that occur after acute bacterial infection [24]. The cellular immune response following infection and treatment was determined by measuring the gene expression of cytokines. Earlier studies confirmed the expression of various cytokines in *Salmonella*-infected intestinal epithelial cells [24, 47]. Treatment of infected cells with MT up-regulated the gene expressions of IL-6, IL-8 and IL-10. Activation of these cytokines is crucial for initiating the host-defense against *S*. Typhimurium. Inflammatory bowel disease was observed in mice deficient with IL-10 [48]. The up-regulation of IL-6 and IL-8 is critical for the host because of high biological significance. Indeed, IL-6 plays a critical role in regulation of hematopoiesis, inflammation and immunity [49]. It is also correlated with the differentiation of B cells and macrophages. Likewise, IL-8 is a pro-inflammatory chemokine that attracts and activates neutrophils to the intestinal lumen [50].

## Conclusions

Our findings suggest that MG enhances the membrane-destabilizing and biofilm inhibitory effects of Ty in *S*. Typhimurium. Sub-inhibitory concentrations of MT were also effective in reducing the adhesion and invasion of *S*. Typhimurium to intestinal epithelial cells. The direct anti-bacterial activity and indirect stimulation of the host response could be the mechanism of anti-*Salmonella* activity of MT. Therefore, co-administration of MG with Ty could be considered to reduce the fecal burden of *S*. Typhimurium in swine and thereby reduce transmission of infection to humans. Future work will aim at evaluating the *in vivo* efficacy of MT in target animal.

## Supporting information

S1 Table List of primers used for qRT–PCR. (DOCX)

S1 Fig Crystal violet staining of *S*. Typhimurium (ATCC 14028) biofilm in the presence of methyl gallate (MG) (**A**) and tylosin (Ty) (**B**). The effect was not significantly different in *S*. Typhimurium LVPP-STI15 biofilm. Values represent mean ± SEM of four independent experiments.
(JPG)

S2 Fig **Cytokine expression of IL-8 (A), IL-10 (B), IL-6 (C), IL-1β (D) and TNFα (E) in Caco-2 monolayers co-cultured with methyl gallate (MG), tylosin (Ty) and various concentrations of their combination (MT) during 8 h infection with *S*. Typhimurium.** Cell: non-infected and non-treated control. *Salmonella*: infected, but non-treated cells. Results mean ± SEM of three independent experiments. MG = 32 μg/mL; Ty = 64 μg/mL; ^1^/_16_× MT = 8 μg/mL MG and 16 μg/mL Ty; ^1^/_8_× MT = 16 μg/mL MG and 32 μg/mL Ty; and ¼× MT = 32 μg/mL MG and 64 μg/mL Ty. *P < 0.05 compared to infected, but non-treated cells; ^τ^*P* < 0.05 compared to Ty; ^π^*P* < 0.05 compared to MG; and ^#^*P* < 0.05 compared to both MG and Ty.
(JPG)

## Abbreviations

ANOVA: analysis of variance
CFU: colony-forming unit
CV: crystal violet
EC_50_: concentration that results in 50% inhibition of the maximum biofilm formation
FBS: foetal bovine serum
FICI: fractional inhibitory concentration index
GPx: glutathione peroxidase
IL: interleukin
LB: Luria–Bertani
MBC: minimum bactericidal concentration
MEM: minimum essential medium
MG: methyl gallate
MHB: Mueller–Hinton broth
MIC: minimum inhibitory concentration
MP: membrane potential
MT: combination of methyl gallate (MG) and tylosin (Ty)
MTT: 3-(4,5-dimethyl-2-thiazolyl)-2,5-diphenyl-2H-tetrazolium bromide
PBS: phosphate-buffered saline
PG: pyrogallol
PI: propidium iodide
qRT–PCR: quantitative real-time reverse transcription PCR
RPMI: Roswell Park Memorial Institute
SEM: scanning electron microscope
Ty: tylosin
TNFα: tumour necrosis factor alpha
