# Supplementary material for: Methyl gallate and tylosin synergistically reduce the membrane integrity and intracellular survival of Salmonella Typhimurium
Source: PLoS One. 2019 Sep 6;14(9):e0221386. doi: 10.1371/journal.pone.0221386 (PMC6730861; doi:10.1371/journal.pone.0221386)
Supplement: S1 Table — (DOCX) [file pone.0221386.s001.docx]

**S1 Table.** List of primers used for qRT–PCR

| Target gene | Primer sequence | Accession number |
| --- | --- | --- |
| IL-6 | F-5′- GGTACATCCTCGACGGCATC-3′  R-5′- GCCTCTTTGCTGCTTTCACAC-3′ | NM_000600 |
| Tnf-α | F-5′- TGGAGAAGGGTGACCGACTC-3′  R-5′- TCCTCACAGGGCAATGATCC-3′ | NM_000594 |
| IL-8 | F-5′-AAACCACCGGAAGGAACCAT-3′  R-5′-GCCAGCTTGGAAGTCATGT-3′ | NM_000584 |
| IL-1β | F-5′- CTGTACGATCACTGAACTGC-3′  R-5′-CACCACTTGTTGCTCCATACT-3′ | NM-000576 |
| β-actin | F-5′-CTTCTACAATGAGCTGCGTG-3′  R-5′-CATGAGGTAGTCAGTCAGG-3′ | NM_001101 |
| IL-10- | F-5′-GGGAGAACCTGAAGACCCTCA-3′  R-5′-TGCTCTTGTTTTCACAGGGAAG-3′ | NM_000572 |
| GAPDH | F-5’ -GACCACAGTCCATGCCATCA-3’  R-5’ -CATCACGCCACAGTTTCCC-3’ | NM_002046 |

(F= forward primer; R= reverse primer)

GADPH: Glyceraldehyde-3-phosphate dehydrogenase
