# Supplementary figures and images for: Methyl gallate and tylosin synergistically reduce the membrane integrity and intracellular survival of Salmonella Typhimurium
Source: PLoS One. 2019 Sep 6;14(9):e0221386. doi: 10.1371/journal.pone.0221386 (PMC6730861; doi:10.1371/journal.pone.0221386)

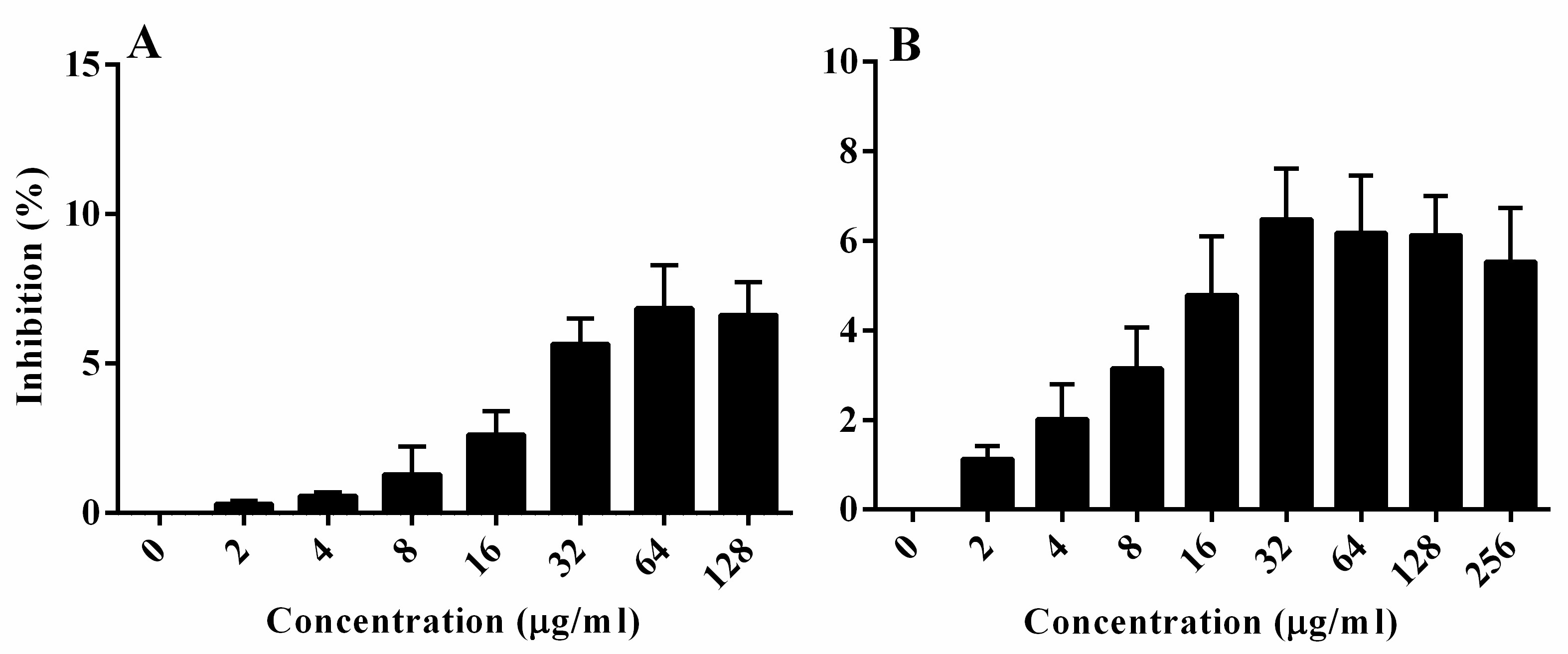

Supplement: S1 Fig — Crystal violet staining of S. Typhimurium (ATCC 14028) biofilm in the presence of methyl gallate (MG) (A) and tylosin (Ty) (B). The effect was not significantly different in S. Typhimurium LVPP-STI15 biofilm. Values represent mean ± SEM of four independent experiments. (JPG) [file pone.0221386.s002.jpg]

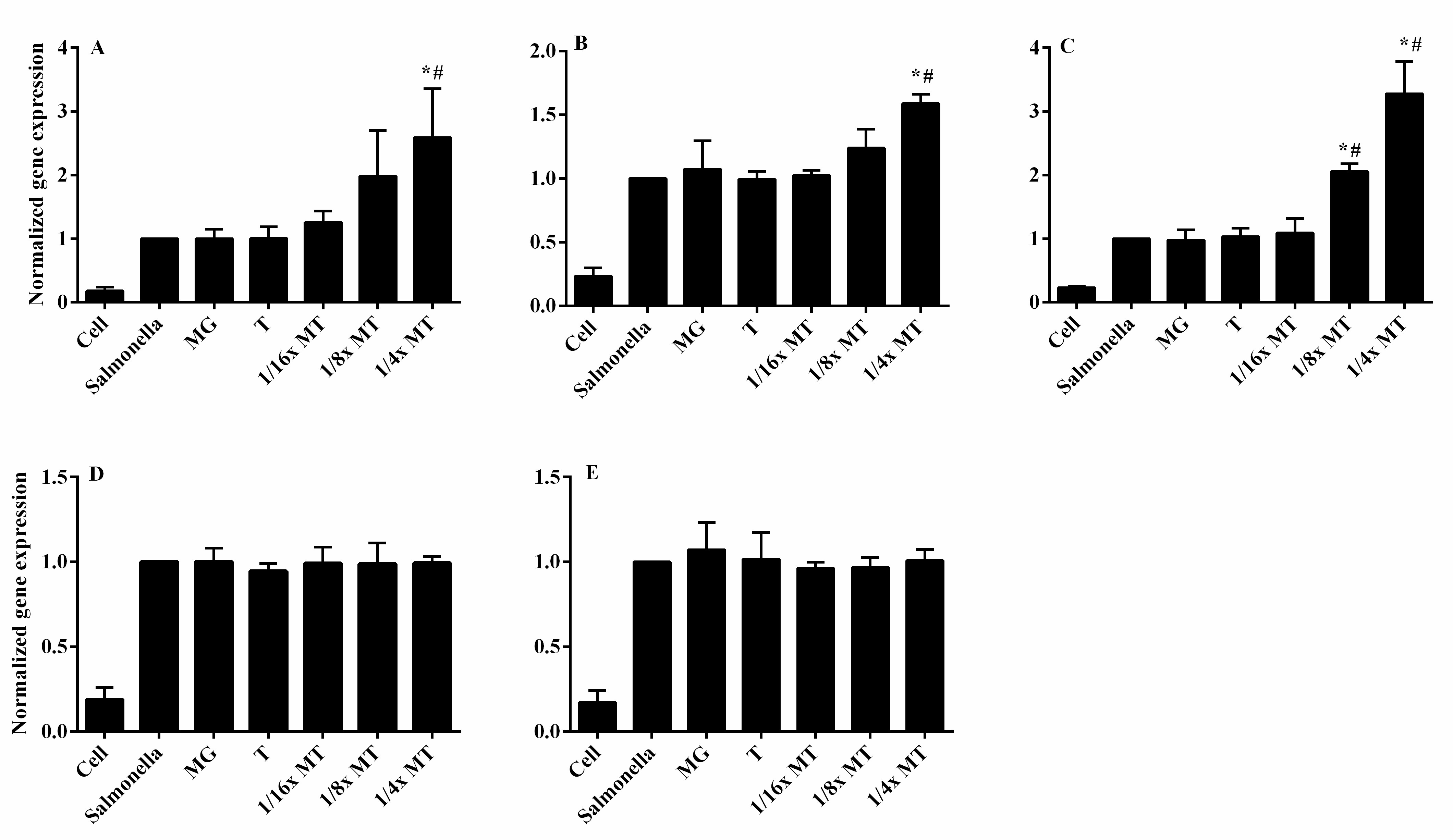

Supplement: S2 Fig — Cytokine expression of IL-8 (A), IL-10 (B), IL-6 (C), IL-1β (D) and TNFα (E) in Caco-2 monolayers co-cultured with methyl gallate (MG), tylosin (Ty) and various concentrations of their combination (MT) during 8 h infection with S. Typhimurium. Cell: non-infected and non-treated control. Salmonella: infected, but non-treated cells. Results mean ± SEM of three independent experiments. MG = 32 μg/mL; Ty = 64 μg/mL; 1/16× MT = 8 μg/mL MG and 16 μg/mL Ty; 1/8× MT = 16 μg/mL MG and 32 μg/mL Ty; and ¼× MT = 32 μg/mL MG and 64 μg/mL Ty. *P < 0.05 compared to infected, but non-treated cells; τP < 0.05 compared to Ty; πP < 0.05 compared to MG; and #P < 0.05 compared to both MG and Ty. (JPG) [file pone.0221386.s003.jpg]
